# Dimensional Accuracy of Intraoral Scanners in Recording Digital Impressions of Post and Core Preparations: A Systematic Review

**DOI:** 10.3390/diagnostics14242890

**Published:** 2024-12-23

**Authors:** Saeed M. Alqahtani, Mohammed Salman Almalki, Mai Almarzouki, Saad Saleh AlResayes, Nisreen Nabiel Hassan, Arwa Jaber I. Mohana, Majed S. Altoman, Mohammed E. Sayed

**Affiliations:** 1 Department of Prosthetic Dentistry, College of Dentistry, King Khalid University, Abha 62529, Saudi Arabia; smaalqahtani@kku.edu.sa (S.M.A.); arwaj.mohanna@gmail.com (A.J.I.M.); maltoman@kku.edu.sa (M.S.A.); 2 Department of Prosthodontics, Ministry of Health Saudi Arabia, Abha 62461, Saudi Arabia; almalki.dent@gmail.com; 3 Department of Restorative Dentistry, Faculty of Dentistry, King Abdulaziz University, Jeddah 21589, Saudi Arabia; mzalmarzouki@kau.edu.sa; 4 Department of Prosthetic Dental Sciences, College of Dentistry, King Saud University, Riyadh 11451, Saudi Arabia; salresayes@ksu.edu.sa; 5 Restorative Dental Science Department, Taibah University, Madina 42353, Saudi Arabia; nnhassan@taibahu.edu.sa; 6 Department of Prosthetic Dental Sciences, College of Dentistry, Jazan University, Jazan 45142, Saudi Arabia

**Keywords:** CAD/CAM, intraoral scanner, lab scanner, digital impression, post and core, accuracy, trueness, precision, root canal preparation

## Abstract

Background: This study aims to perform a review by selecting, analyzing, and evaluating articles that discuss the accuracy of intraoral scanners (IOSs) in recording post space compared to conventional impression-making techniques. Methods: The review question framed using the PITR framework (participant, index test, targeted condition, and reference standard) is as follows: What is the dimensional accuracy (T) of impressions made using intraoral scanners (I) for post space (P) compared to impressions made using conventional techniques and digitalized using extraoral scanners (R)? Four electronic databases were searched using pre-set keywords. The guidelines and strategies recommended by PRISMA formed the basis for planning, executing, and documenting this systematic review. QUADAS-2 was used to critically analyze the quality of all the selected articles. Results: After excluding ineligible articles, the end synthesis has nine studies (*n* = 9) for qualitative analysis. All nine evaluated studies were found to be at risk of bias, with high or unclear risk in one or more domains. Three out of nine evaluated studies had unclear concerns regarding the applicability, and the remaining six had low concerns. In all the included studies, the IOSs were reported to have deviations in accuracy compared to the conventional techniques for making digital impressions of post space. Conclusions: The accuracy of IOSs was found to be inversely proportional to the length of post space and directly proportional to the diameter of post space. IOSs, when used adequately in short post spaces, can be an alternative to conventional impression-making for making custom posts and cores.

## 1. Introduction

The advent of new technologies and machines has helped dentists perform more accurate diagnosis and efficient treatment planning and provide superior treatment quality to their patients in a shorter duration of time [1,2,3,4]. A surge in interest in digitalization and the use of Computer-Aided Designing and Computer-Aided Machining (CAD/CAM) in restorative dentistry has opened new potentials for restorations by minimizing operator errors, increasing accuracy, and reducing the time and fabrication cost of the prosthesis [5,6,7].

The treatment of choice for the management of endodontically treated teeth with minimal coronal tooth structure involves restoring the tooth with post and core prosthetic restoration [8]. Post and core can be either prefabricated or custom-made, each with merits and demerits. Prefabricated posts are commonly used as they are easier to place, come in tooth-colored materials, and have elasticity similar to dentine, thus minimizing the risk of tooth fracture [9,10,11]. Their drawback involves inadequate fit in the root canal, causing an increase in luting cement thickness, leading to stress in cement and subsequent fractures in cement, and eventually leading to poor retention of the post [12,13,14]. Additionally, canal preparation leads to more dentine removal, and restoration failure is possible at the junction of the post and core [15]. Custom-made posts and cores are still the preferred treatment for restoring these teeth as they adapt well to the root canal anatomy [15,16]. With the incorporation of CAD/CAM in the field of restorative dentistry, dentists have the option of using various non-metallic materials for the fabrication of custom posts and cores, thus overcoming various shortcomings of traditional metallic custom posts and cores like frequent root fracture and non-esthetics [17,18]. Digital impressions made by intraoral scanners (IOSs) can be repeated easily and transferred to production laboratories, thus saving time, costs, and errors related to material deformations [19,20,21,22]. The drawbacks include the high initial cost of the instrument, difficulties in recording in the presence of oral fluids, and challenges in recording deeper areas [23]. 

The success of any indirect prosthetic restoration hinges on the precision of registration. This is particularly crucial for custom posts and cores, where accurate recording of the canal morphology is essential for a precise fit of the post in the root canal. Traditional methods of recording post space involve making impressions with polyvinyl siloxane impression materials or using pattern resins directly [24,25,26]. However, the use of scanners to make digital impressions is gaining popularity. These scanners can digitize impressions in the lab or be used intraorally, eliminating the need for impression materials. IOSs, commonly used for recording impressions of extra coronal structures, have been shown to provide restorations with high accuracy [27,28,29,30]. With ongoing advancements in this technology, they are becoming more user-friendly and their accuracy levels are improving, matching those of conventional impression materials and techniques. 

To reduce chair-side time and to improve patient compliance, these IOSs are now used to record post space of the endodontically treated teeth. IOSs work on the principle that light beams emitted by the scanner should reach the target areas, and then, the reflected beams should be recorded back by the scanner to build a digital image of the object [31,32,33,34]. Recording post space in the root canal with IOSs is challenging due to the limited diameter and greater depth of the canal. With the availability of new generations of IOSs, studies have reported acceptable accuracy levels of digital impressions made for posts and cores [12,35,36,37]. Studies comparing the accuracy of IOSs in recording post space with different depths and diameters have shown varying results [5,12,35,36,37,38,39,40,41].

To our knowledge, there are no systematic reviews that have assessed the accuracy of IOSs in recording post space compared to conventional techniques. Thus, the current study aims to perform a review by selecting, analyzing, and evaluating the articles discussing the accuracy of IOSs in recording post space compared to conventional impression-making techniques. In this review, we address the following question: Do post space impressions made using intraoral scanners have the same accuracy compared to impressions made using conventional techniques and digitalized using extraoral scanners?

## 2. Materials and Methods

The present systematic review was registered in the PROSPERO register (No. CRD42024536149). The guidelines and strategies recommended by Preferred Reporting Items for Systematic Reviews and Meta-Analyses (PRISMA) [42] formed the basis for planning, executing, and documenting this systematic review. Four electronic databases were searched on the 13th and 14th of March 2024 and were later updated on the 30th and 31st of July 2024. The article search was limited to articles published after January 2000. The articles were evaluated based on the inclusion and exclusion criteria presented in Table 1.

### 2.1. Selection Criteria

Table 1 summarizes the criteria used for selecting the research articles in the current systematic review.

### 2.2. Exposure and Outcome

In the present systematic review, the relevant exposure is the impression of post space made using different IOSs, irrespective of the type of IOS, whereas the outcome of the interest is the accuracy assessment of these IOSs in recording the post space impression compared to digitalized conventional techniques. The review question framed using the PITR framework [43] (participant, index test, targeted condition, and reference standard) is as follows: What is the dimensional accuracy (T) of impressions made using intraoral scanners (I) for post space (P) compared to impressions made using conventional techniques and digitalized using extraoral scanners (R)?

### 2.3. Search Strategy, Study Selection, and Data Extraction

Four electronic databases (PubMed, Web of Science, Scopus, and Cochrane) were searched systematically. The keywords employed for the search were ‘Post Space’ AND ‘intraoral scanner impression’ AND ‘conventional impression technique’ AND ‘Accuracy’. In April 2024, two independent authors (M.E.S. and S.M.A.) used the listed search strings, Boolean operators, and truncation and performed the search in these electronic databases. The search strings were modified as per the necessities of the particular database (Table 2). After removing the duplicate articles, M.E.S. and S.M.A. independently reviewed the titles, abstracts, and articles and excluded those that did not meet the selection criteria. The references within the shortlisted articles were searched manually to avoid missing any pertinent articles. M.S.A. and M.A. later independently reviewed the full texts of these selected articles and tested their eligibility for selection. The shortlisted articles were rechecked by M.E.S. and M.A., and any disagreements between the authors were settled by discussion amongst all four authors (M.E.S., M.A., M.S.A., and S.M.A.). 

M.E.S., S.S.A., and N.N.H. prepared a self-designed data extraction chart to list the relevant findings of the selected articles. The information was listed under the following subheadings: author, year, and country; study type; sample size (*n*); teeth evaluated; post space length and diameter; intraoral scanner manufacturer and model no. used; comparator/control; accuracy measurement areas; accuracy parameters tested; use of Scan Post; technique of testing accuracy/depth; 3D matching/evaluation software program used; deviations/discrepancy/space; and authors’ suggestions/conclusions of the study.

### 2.4. Quality Analysis of the Included Studies

The quality of the articles included in this review was critically analyzed using the Quality Assessment and Diagnostic Accuracy Tool (QUADAS-2) [44,45]. It effectively evaluates both the risk of bias and applicability concerns. The risk of bias component consists of four key domains, namely, patient selection, index test, reference standard, and flow and timing, whereas, the applicability concern component includes three key domains: patient selection, index test, and reference standards.

## 3. Results

**Figure 1:**
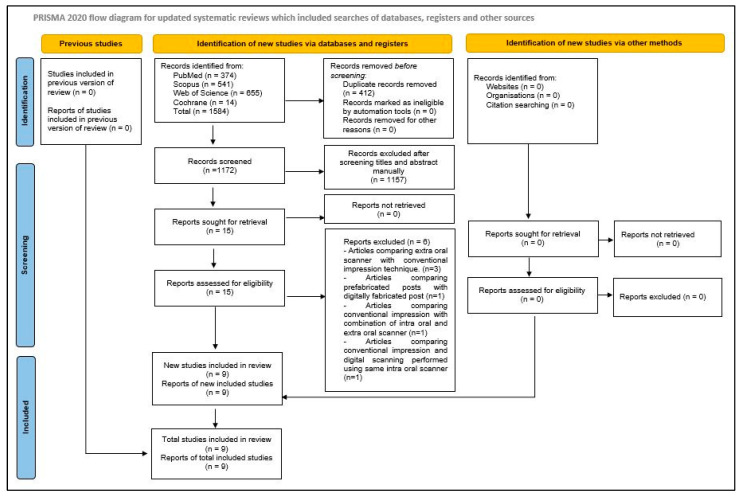
Article selection strategy based on PRISMA guidelines.

A preliminary electronic data search resulted in 1584 hits, of which 412 records were observed to be duplicated and removed. The titles and abstracts of the remaining 1172 records were reviewed, of which 1157 were omitted as they did not meet the selection criteria. Fifteen studies were evaluated for eligibility. The bibliography of these fifteen studies (*n* = 15) was searched manually for any additional articles, but no new relevant studies were found. The full text of these fifteen studies was reviewed, and five reports were excluded. In three of the excluded studies, extraoral scanners were compared to conventional impression techniques, whereas another one compared prefabricated posts to digitally fabricated posts and conventional impressions to a combination of intraoral and extraoral scanners. In the final excluded study, the authors performed conventional impressions and digital scanning using the same IOS. So, overall, nine studies (*n* = 9) were selected for qualitative analysis (Figure 1). 

### 3.1. Quality Assessment Outcomes

**Figure 2:**
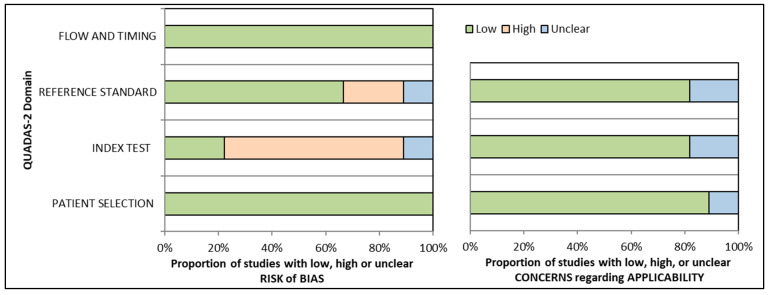
Graphical presentation of QUADAS-2 results.

The Quality Assessment and Diagnostic Accuracy Tool (QUADAS-2) was used to critically analyze the quality of all the selected articles (Figure 2 and Table S1). All nine evaluated studies were found to be at risk of bias, with high or unclear risk in one or more domains. Three out of nine evaluated studies had unclear concerns regarding the applicability, and the remaining six had low concerns. As all the included studies used the same model/teeth for both the index test and the reference standard test, there was a low risk of bias in the data selection domain across all studies. The results from the risk of bias arm demonstrated that 22.22% of the studies had a low risk, 11.11% had an unclear risk, and 66.66% had a high risk in the index test domain. In contrast, in the reference standard domain, 66.66% of the studies had a low risk of bias, 11.11% had an unclear risk of bias, and 22.22% had a high risk of bias. As the same teeth/model were used for both the index test and the reference test and there were no interval or additional interventions between the index and reference standard tests, the flow or time frame was not affected by the final output. Therefore, in all studies, both aspects were regarded as low-risk categories (100%). For the applicability concern arm, 9% had low concerns whereas 11.11% had high concerns in the data selection domain. Additionally, 22.22% had unclear concerns whereas 78.78% had low applicability concerns in both the index test and reference standard domain.

### 3.2. Study Characteristics

**Figure 3:**
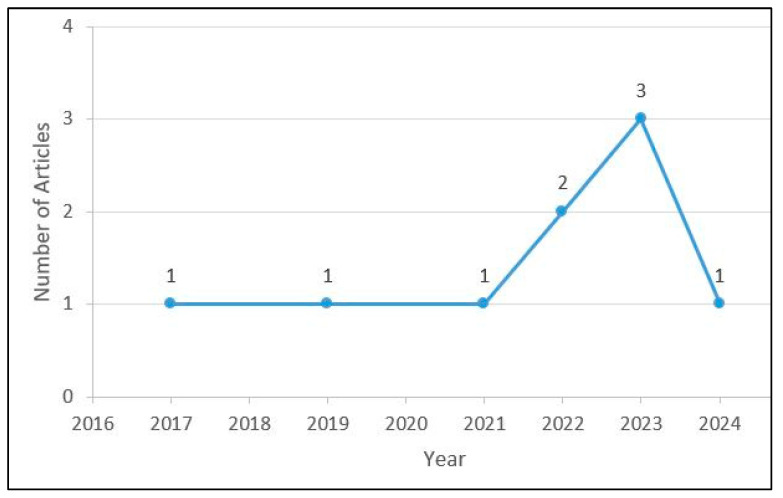
Research trends on IOS use in post and core impressions.

Out of the nine studies included in this review, eight were published in the last five years (between 2019 and 2024) [5,12,35,36,37,38,39,40], whereas one [41] was published seven years ago, in 2017 (Figure 3). Two out of nine studies were conducted in Egypt [12,38], whereas one study each was conducted in Italy [41], Iran [37], Solvenia [5], Germany [36], Turkey [35], France [40], and the USA [39] (Table 3).

Seven studies [5,12,35,36,37,38] used natural extracted human teeth (incisors, canines, or premolars) for their research; one study [39] used typodont maxillary central incisors, and another study [40] used a 3D printed model simulating mandibular premolars. The dimensions of post space prepared in these teeth varied amongst the included studies. The post space length varied from 6 mm [5,39] to 8 mm [12,38,39], 8.5–9.8 mm [41], 10 mm [35,37,38,39,40], 12 mm [35,36], 14–20 mm [35]. The diameter of the prepared canals also varied from 0.9 mm [5,36,37] to a maximum of 3 mm [12,40]. The type of IOS used varied in the selected studies. The three most commonly used IOSs were Primescan [12,35,36,38,39,40], Trios 3Shape [5,12,36,37,40,41], and Medit [12,38,40], with variations in the model and version of the scanner. A lab scanner was used as a comparator by seven studies (an InEos X5 lab scanner [12,35,38,39], different models of 3Shape lab scanners [37,40], and a Medit lab scanner [5]). One study used Micro-CT as a comparator [36], whereas one study used a millimeter gauge for comparative measurements [41]. In two studies [12,40], the extraoral scanner was used to scan the post space. In four of the studies [35,38,39,41] conventional silicone impressions were scanned, whereas in two other studies [5,37], conventional cast posts were scanned for comparison. Only three studies [5,36,37] used Scan Posts to record the post space. Most of the studies performed superimposition techniques using either Geomagic^®^ Control X™ [12,35,38,39] or GOM Inspect [5,36,40] 3D matching software to evaluate accuracy. The included studies measured the accuracy of IOSs as either trueness in terms of space volume in Root Mean Square (RMS) or linear discrepancy (post-scan length vs. space depth) (Table 3).

### 3.3. Features of the Included Studies

(A) Accuracy (space volume in RMS):

When comparing trueness, all studies reported deviations in recording post space by IOSs compared to extraoral scanners.

(a) Accuracy with a post length of 8 mm:

Taha et al. [12] reported higher accuracy with an increase in the cervical diameter of the post space. Three IOSs were used, namely, Primescan, Medit i600, and Trios 3Shape; the reported deviations in RMS at a cervical diameter of 2.5 mm were 36.21 ± 4.36 μm, 85.35 ± 5.46 μm, and 86.08 ± 2.50 μm and deviations in RMS at a cervical diameter of 3 mm were 38.27 ± 4.93 μm, 37.48 ± 10.37 μm, and 39.55 ± 4.49 μm, respectively. In the study by Emama et al. [38], with a post space diameter of 1.6 mm, the reported deviations in RMS were 330 ± 90 μm for Primescan IOS, 180 ± 30 μm for Medit i500, and 310 ± 70 μm for Carestream 3600 IOS. Almalki et al. [3] reported the deviation in RMS to be 81 µm when using CEREC IOS at a post space diameter of 1.3 mm.

(b) Accuracy with a post length of 10 mm:

In a study by Emma et al. [38], the deviations in RMS were reported to be 200 ± 70 μm for Primescan IOS, 180 ± 60 μm for Medit i500, and 260 ± 1100 μm for Carestream 3600 IOS. Almalki et al. [39] reported a deviation of 101 µm when using Ceres IOS, whereas a higher deviation of 357.1 μm was reported by Elter et al. [35] when they used CEREC Primescan 5.0.0 IOS. They also reported that as the depth of post space increases, the deviation increases from 357.1 μm at a 10 mm depth to 897.5 μm at a 20 mm depth.

(B) Linear discrepancy (post-scan length vs. space depth)/apical gap:

Emam et al. [38] reported mean apical gaps of 40 µm, 30 µm, and 1420 µm when using Primescan, Medit i500, and Care stream IOS, respectively, at a post length of 8 mm, whereas with a post length of 10 mm, the readings reported were higher: 120 µm, 1110 µm, and 3530 µm, respectively. Hendi et al. [37] reported an apical gap of 290 ± 120 µm when using Trios 3Shape IOS for a post space of 10 mm. In contrast, Leven et al. [36] reported an apical linear discrepancy of 55 µm with both Primescan and Trios 3Shape IOS at a post space of 12 mm. These values were lower (51.25 µm) when Scan Posts were used for scanning.

## 4. Discussion

Constant advancements in the field of IOSs are helping dentists provide enhanced treatment quality to patients. However, the use of these IOSs in recording post space is still debatable due to accuracy issues. 

Here, we review relevant articles in this area of research and discuss the accuracy of intraoral scanners in recording post space compared to conventional techniques. To our knowledge, this is the first review to analyze these findings. All nine articles included in this review are in vitro studies. Four articles [35,37,38,39] discussed the accuracy of tested IOSs compared to conventional silicone impressions scanned using a lab scanner. Three studies compared the accuracy of conventional cast post (fabricated using pattern resin) to that of scans obtained with a lab scanner [5], a Micro-CT dataset [36], or conventional silicone impressions and post length measured with a millimeter gauge [41]. Two studies [12,40] compared the accuracy of the data obtained when post space was directly scanned with a lab scanner. The systematic review’s outcome suggests that IOSs’ accuracy in recording post space is inversely proportional to post space depth; as the post space depth increases, the accuracy decreases. The outcomes based on the nine articles suggest that the use of IOSs in making post space impressions is an acceptable option, with certain limitations in the post space length and diameter to be recorded. The accuracy of various IOSs in recording different post space lengths will be discussed below.

In all the included studies, the IOSs were reported to have deviations in accuracy compared to the control group. The studies reported contrasting results regarding the accuracy of the IOSs used. When comparing Primescan, Medit i600, and Trios 3Shape IOSs for the measurement of an 8 mm post length, Taha et al. [12] reported the highest trueness for Primescan, followed by Medit, with the lowest value for Trios 3Shape IOS. In the study by Emmam et al. [38], Medit i500 showed better accuracy, followed by Carestream and Trios 3Shape IOS, when used to measure post spaces of 8 mm and 10 mm. When comparing Primescan, Omnicam, Medit i700, and TRIOS 4, 3Shape A/S for the measurement of a 10 mm post space, Duapagne et al. [40] reported differences between scanners, with the lowest error reported by Primescan IOS. Leven et al. [36] reported the lowest linear discrepancy by Trios 4 when used with Scan Post, followed by Trios 4 without Scan Post and Primescan IOS when used for a 12 mm post space. 

The studies tested the accuracy of various IOSs that work on different imaging concepts, although the manufacturing companies did not describe them clearly due to patent restrictions. The difference in the accuracy of these scanners can be attributed to the following [5,12,35,41]. Primescan uses structured light-confocal microscopy technology with an intelligent pixel sensor as well as a high-frequency contrast analysis scanning concept. Trios also uses light-confocal microscopy, along with ultrafast optical scanning methods. Medit uses a different concept, i.e., video-type scanning based on optical triangulation and image stitching. CS3600 also employs video-type scanning based on optical triangulation but uses a video sequence system of image processing. [12,38,39,46]. The literature contains contrasting reports, with some claiming better accuracy of IOSs working on principles of active triangulation compared to confocal microscopy, stating that data acquired by video sequencing have higher trueness than data acquired by individual imaging [47,48]. In contrast, other studies reported different results [49,50]. All IOSs work on the concept that an adequate quantity of light beams must be projected to the area of interest, and later, these light beams should be captured and recorded by the scanner after they are reflected back. Therefore, IOSs with larger capture boxes allow light beams to reach deeper post spaces compared to IOSs with smaller capture boxes, requiring greater stitching and linking of picture records. These can lead to higher error incorporation [38,40].

Seven studies used an extraoral scanner (InEos X5/3Shape/Medit) as a reference scanner for evaluating the overall deviation in accuracy by superimposition [5,12,35,37,38,39,40]. The studies reported high accuracy of these scanners, with a deviation of 15 μm [5,12,32,38,51]. One study [36] used Micro-CT for comparison, which has been used in various studies before [52,53,54]. A study by Pinto et al. [41] used manual measurements, which may not be accurate to the level of micromillimeters. Four of the included studies that performed superimposition for accuracy evaluation used Geomagic Control XReverse engineering software [12,35,38,39], whereas three other studies [5,36,40] used GOM 3D analysis software. Both software were reported to have good accuracy [55]. 

The length and diameter of the post space play an important role in the accuracy of IOSs. Most of the included studies reported that as the length increases, the accuracy of recording decreases, whereas with an increase in diameter, the accuracy of most of the IOSs increases. This is related to the scanner’s ability to project an adequate quantity of light beams to the area of interest and record the reflected beams. The accuracy of IOSs is considered clinically acceptable if the deviation value is below 120 μm [56,57,58,59]. This applies especially to scanning prepared teeth as the acceptable threshold for the marginal gap for a crown should be less than 120 μm. However, for post space impressions, the parameters can be set differently. For post space, a cement thickness of 250–500 μm was reported to be clinically acceptable [35,60,61]. So, IOSs that show a deviation value less than this can be deemed clinically acceptable. The studies included in this review reported deviations in accuracy values that were within the clinically acceptable range of 250–500 μm when the post space length is up to 14 mm [5,12,35,37,38,39,40] and the diameter is greater than 2.2 mm [12,35]. This deviation increased when the post space length increased and the diameter decreased. Some studies reported minimal deviations in accuracy values in the cervical and middle region of the post space [5,36,39,40].

Various other factors can affect the accuracy of IOSs, including the presence of adjacent teeth. Dupagne et al. [40] reported that the presence of adjacent teeth decreases the accuracy of the recording of post space by IOSs. The use of Scan Post has also been reported to affect accuracy. Leven et al. [36] reported higher deviation values in recording post space when Scan Posts are used for recording. This was attributed to the fact that Scan Posts are held in the canal by friction and do not correspond precisely to the preparation drill, leading to higher inaccuracies [5]. Operators’ skills, illumination, and scanning techniques also affect the accuracy of post space impressions while using IOSs [35,37,38,39].

The results of this systematic review are also related to the different generations of scanners and methods of recording and analyzing the data used by the included studies. Older studies [37,41] used ruler tools or millimeter gauges to measure the discrepancy, whereas newer studies used superimposition techniques and measured variations in accuracy using 3D analysis software. Additionally, the use of newer generations of IOSs in recent studies adds heterogeneity to this systematic review. Only two studies used Scan Post to record post space. The results of this review are vital as they can guide dentists in decision-making regarding the selection of the best impression-making technique and choosing the suitable IOS for recording post space in endodontically treated teeth, thus improving the accuracy of the impressions and reducing the failure rate of the post and core.

## 5. Strength and Limitations

A systematic comprehensive search strategy and robust methodology are the key highlights of this systematic review. The article search and data extraction were conducted independently by multiple authors, thus ensuring that no relevant article was excluded and that all the relevant data were tabulated for discussion. All articles comparing the accuracy of IOSs to other conventional methods of recording post space were evaluated for inclusion. Bias was controlled by involving several authors in selecting the articles that met the eligibility criteria for inclusion. The limitations include a study search that was restricted to English-language articles, articles published after January 2000, and studies performed in vitro only (as no in vivo study could meet the inclusion criteria), where it was not possible to create actual oral conditions. Additionally, most of the included studies were heterogeneous and of medium to high quality, but they had a greater risk of bias. Quantitative analysis of the included studies was attempted, but due to the heterogeneous nature of the included studies, as determined by a meta-analysis, the outcomes of pooled estimates were inconclusive. So, it was not included in the present study. We suggest standardizing the methodology (specimen preparation and tests) of in vitro studies and data availability in both graphical and tabular forms in the result section of published articles for ease of utilization. 

## 6. Conclusions

Within the limitations of this review, the following conclusions can be drawn:

- IOSs have more variations in accuracy than conventional techniques when making digital impressions of post space. These values were within the clinically acceptable range when post space lengths up to 14 mm were recorded.
- The accuracy of IOSs is inversely proportional to the length of post space and directly proportional to the diameter of post space.
- Variations in accuracy are higher in apical regions compared to the cervical and middle third regions.
- IOSs, when used adequately in short post spaces, can be an alternative to conventional impression-making for making custom posts and cores.

## 7. Future Directions

More in vivo studies should be conducted to assess the effectiveness of intraoral scanners (IOSs) in capturing impressions of post space areas. Additionally, manufacturers should focus on innovating IOSs by optimizing the size of the scanner’s tip, improving the management of optical rays, and creating scan bodies that are compatible with post space drill systems.

## Figures and Tables

**Table 1:** Article selection criteria.

Inclusion Criteria	Exclusion Criteria
Articles published in the English language	Articles published in a language other than English
Human studies	Animal studies
Articles published between January 2000 and July 2024	Articles published before January 2000
In vitro and in vivo studies, with no limitations on the research design	Opinions, commentaries, editorials, case reports, reviews, unpublished abstracts, unfinished trials, review papers, and dissertations
Studies evaluating the accuracy of IOSs in making impressions of post space compared to other impression-making techniques	Studies evaluating the accuracy of intraoral scanners in making impressions of other intraoral structures
	Studies discussing IOSs under trial
	Studies comparing the accuracy of different extraoral scanners

**Table 2:** Search strings and strategy.

**Database**	**Combination of Search Terms and Strategy**	**Number** **of** **Titles**
MEDLINE/PubMed	((“post and core technique”[MeSH Terms] OR “root canal preparation”[MeSH Terms] OR “computer aided design”[MeSH Terms] OR “diagnosis, computer assisted”[MeSH Terms] OR “dental dowel *”[Title/Abstract] OR “post and core”[Title/Abstract] OR “endodontically treated teeth”[Title/Abstract] OR “post space”[Title/Abstract] OR “anatomic post”[Title/Abstract] OR “custom post and core”[Title/Abstract] OR “CAD-CAM”[Title/Abstract] OR “CAD-CAM”[Title/Abstract] OR “digital dentistry”[Title/Abstract] OR “digital workflow”[Title/Abstract] OR “computer aided manufacturing”[Title/Abstract] OR “computer assisted manufacturing”[Title/Abstract] OR (“post”[All Fields] AND “space depth”[Title/Abstract])) AND (“intraoral scanner”[Title/Abstract] OR “digital impression”[Title/Abstract] OR “scan post”[Title/Abstract] OR “post space impression”[Title/Abstract] OR “imaging three dimensional”[Title/Abstract]) AND (“dental impression technique”[MeSH Terms] OR “dental impression materials”[MeSH Terms] OR “X ray microtomography”[MeSH Terms] OR “extra oral scanner”[Title/Abstract] OR “lab scanner”[Title/Abstract] OR “benchtop scanner”[Title/Abstract] OR “conventional impression”[Title/Abstract] OR “X ray micro computed tomography”[Title/Abstract] OR “X ray micro ct”[Title/Abstract] OR “elastomeric impression”[Title/Abstract] OR “addition silicone impression”[Title/Abstract]) AND (“dimensional measurement accuracy”[MeSH Terms] OR (“accuracies”[All Fields] OR “accuracy”[All Fields]) OR (“precise”[All Fields] OR “precised”[All Fields] OR “precisely”[All Fields] OR “preciseness”[All Fields] OR “precises”[All Fields] OR “precision”[All Fields] OR “precisions”[All Fields]) OR “trueness”[All Fields] OR ((“measurability”[All Fields] OR “measurable”[All Fields] OR “measurably”[All Fields] OR “measure s”[All Fields] OR “measureable”[All Fields] OR “measured”[All Fields] OR “measurement”[All Fields] OR “measurement s”[All Fields] OR “measurements”[All Fields] OR “measurer”[All Fields] OR “measurers”[All Fields] OR “measuring”[All Fields] OR “measurings”[All Fields] OR “measurment”[All Fields] OR “measurments”[All Fields] OR “weights and measures”[MeSH Terms] OR (“weights”[All Fields] AND “measures”[All Fields]) OR “weights and measures”[All Fields] OR “measure”[All Fields] OR “measures”[All Fields]) AND (“error”[All Fields] OR “error s”[All Fields] OR “errorful”[All Fields] OR “errors”[All Fields])) OR ((“linear”[All Fields] OR “linearities”[All Fields] OR “linearity”[All Fields] OR “linearization”[All Fields] OR “linearizations”[All Fields] OR “linearize”[All Fields] OR “linearized”[All Fields] OR “linearizer”[All Fields] OR “linearizes”[All Fields] OR “linearizing”[All Fields] OR “linears”[All Fields]) AND (“deviate”[All Fields] OR “deviated”[All Fields] OR “deviates”[All Fields] OR “deviating”[All Fields] OR “deviation”[All Fields] OR “deviational”[All Fields] OR “deviations”[All Fields])))) AND ((2000/1/1:2024/7/31[pdat]) AND (english[Filter]))	374
Web of Science (Core collection)	#1 (P) TS = (‘Post and Core Technique’ OR ‘Root canal preparation’ OR ‘Computer-Aided Design’ OR ‘Diagnosis, Computer-Assisted’ OR ‘Dental dowel *’ OR ‘Post and core’ OR ‘Endodontically-Treated Teeth’ OR ‘Post space’ OR ‘Anatomic post’ OR ‘Custom post and core’ OR CAD-CAM OR CAD/CAM OR ‘Digital dentistry’ OR ‘Digital workflow’ OR ‘Computer-Aided Manufacturing’ OR ‘Computer-Assisted Manufacturing’ OR ‘Post space depth’)) #2 (I) TS = (‘Intraoral scanner’ OR ‘Digital impression’ OR ‘Scan post’ OR ‘Post space impression’ OR ‘Imaging, Three-Dimensional’)) #3 (R) TS = (‘Dental Impression Technique’ OR ‘Dental Impression Materials’ OR ‘X-Ray, Microtomography’ OR ‘Extra oral scanner’ OR ‘Lab scanner’ OR ‘Benchtop scanner’ OR ‘Conventional impression’ OR ‘X-Ray Micro Computed Tomography’ OR ‘X-ray Micro CT’ OR ‘elastomeric impression’ OR ‘Addition silicone impression’)) #4 (T) TS = (‘Dimensional Measurement Accuracy’ OR Accuracy OR Precision OR trueness OR ‘Measurement error’ OR ‘Linear deviation’) #4 AND #3 AND #2 AND #1 Indexes = SCI-EXPANDED, SSCI, A&HCI, CPCI-S, CPCI-SSH, ESCI, CCR-EXPANDED, IC Timespan = Timespan: 1 January 2000 to 31 July 2024 (Publication Date) and English (Languages)	655
Cochrane	#1: MeSH descriptor: [Post and Core Technique] explode all trees; #2: MeSH descriptor: [Root Canal Preparation] explode all trees; #3: MeSH descriptor: [Computer-Aided Design] explode all trees; #4: MeSH descriptor: [Diagnosis, Computer-Assisted] explode all trees; #5: Dental dowel *; #6: Post and core; #7: Endodontically-Treated Teeth; #8: Post space; #9: Anatomic post; #10: Custom post and core; #11: CAD-CAM; #12: Digital dentistry; #13: Digital workflow; #14: Computer-Aided Manufacturing; #15: Computer-Assisted Manufacturing; #16: Post space depth; #17: Intraoral scanner; #18:Digital impression; #19: Scan post: #20: Post space impression; #21: Imaging, Three-Dimensional; #22: MeSH descriptor: [Dental Impression Technique] explode all trees; #23: MeSH descriptor: [Dental Impression Materials] explode all trees; #24: MeSH descriptor: [X-Ray Microtomography] explode all trees; #25: Extra oral scanner; #26: Lab scanner; #27: Benchtop scanner; #28: Conventional impression; #29: X-Ray Micro Computed Tomography; #30: X-ray Micro CT; #31: elastomeric impression; #32: Addition silicone impression; #33: MeSH descriptor: [Dimensional Measurement Accuracy] explode all trees; #34: Accuracy; #35: Precision; #36: trueness; #37: Measurement error; #38: Linear deviation; #39: #1 OR #2 OR #3 OR #4 OR #5 OR #6 OR #7 OR #8 OR #9 OR #10 OR #11 OR #12 OR #13 #14 OR #15 OR #16; #40: #17 OR #18 OR #19 OR #20 OR #21; #41: #22 OR #23 OR #24 OR #25 #26 OR #27 OR #28 OR #29 OR #30 OR #31 OR #32; #42: #33 OR #34 OR #35 OR #36 OR #37 OR #38; #43: #39 AND #40 AND #41 AND #42; [Custom year range: 2000–2024; Language: English]	14
Scopus	(‘Post and Core Technique’ OR ‘Root canal preparation’ OR ‘Computer-Aided Design’ OR ‘Diagnosis, Computer-Assisted’ OR ‘Dental dowel *’ OR ‘Post and core’ OR ‘Endodontically-Treated Teeth’ OR ‘Post space’ OR ‘Anatomic post’ OR ‘Custom post and core’ OR CAD-CAM OR CAD/CAM OR ‘Digital dentistry’ OR ‘Digital workflow’ OR ‘Computer-Aided Manufacturing’ OR ‘Computer-Assisted Manufacturing’ OR ‘Post space depth’) AND (‘Intraoral scanner’ OR ‘Digital impression’ OR ‘Scan post’ OR ‘Post space impression’ OR ‘Imaging, Three-Dimensional’) AND (‘Dental Impression Technique’ OR ‘Dental Impression Materials’ OR ‘X-Ray, Microtomography’ OR ‘Extra oral scanner’ OR ‘Lab scanner’ OR ‘Benchtop scanner’ OR ‘Conventional impression’ OR ‘X-Ray Micro Computed Tomography’ OR ‘X-ray Micro CT’ OR ‘elastomeric impression’ OR ‘Addition silicone impression’) AND (‘Dimensional Measurement Accuracy’ OR Accuracy OR Precision OR trueness OR ‘Measurement error’ OR ‘Linear deviation’) AND PUBYEAR > 2000 AND PUBYEAR < 2024 AND (LIMIT-TO (SUBJAREA, “DENT”)) AND (LIMIT-TO (DOCTYPE, “ar”)) AND (LIMIT-TO (LANGUAGE, “English”)) AND (LIMIT-TO (SRCTYPE, “j”))	541

*: Truncation, P: Population, I: Intervention, C: Comparator, and O: Outcome.

**Table 3:** Summary of studies included in the systematic review.

Author, Year, and Country	Study Type	Sample Size (*n*)	Teeth Evaluated	Post Space Length and Diameter	IOS Manufacturer and Model No. Used	Comparator/Control	Accuracy Measurement Areas	Accuracy Parameters Tested Precision/Trueness/Internal Adaptation/Apical Gap	Scan Post Used	Technique of Testing Accuracy/Depth	3D Matching/Evaluation Software Program	Deviations/Discrepancy/Space	Authors’ Suggestions/Conclusions
Taha et al., 2024, Egypt [12]	In vitro	18	Natural extracted mandibular second premolars	Length: 8 mm Cervical diameter: (A) 2.5 mm (B) 3 mm	(1) Primescan (Dentsply Sirona, Bensheim, Germany) (2) Medit i600 (Medit Corp., Seoul, South Korea) (3) Trios 3	Scanned post space using a lab scanner (inEos X5, Dentsply Sirona, Charlotte, NC, USA)	-	Trueness	No	Superimposition	Reverse engineering software (Geomagic^®^ Control X™ 2018; 3D Systems Manufacturing, Rock Hill, SC, USA)	Deviations in RMS Cervical diameter: (A) 2.5 mm (1) PS: 36.21 ± 4.36 μm (2) Medit: 85.35 ± 5.46 μm (3) Trios: 86.08 ± 2.50 μm (B) 3 mm (1) PS: 38.27 ± 4.93 μm (2) Medit: 37.48 ± 10.37 μm (3) Trios: 39.55 ± 4.49 μm	Trueness: Primescan > Medit i600 > Trios 3
Emam et al., 2023, Egypt [38]	In vitro	16	Natural extracted maxillary central incisors and mandibular premolars	Length: (A) 8 mm (B) 10 mm Diameter: 1.6mm	(1) Primescan AC with Connect™ Software, 4.5 (Dentsply Sirona, Bensheim, Germany) (2) Medit i500 (Medit Corp., Seoul, South Korea) (3) CS 3600 (Carestream Dental, Stuttgart, Germany)	Conventional silicone impressions scanned using a lab scanner (inEos X5, Dentsply Sirona)	-	Trueness and recording depth	No	Superimposition	Reverse engineering software (Geomagic^®^ Control X™ 2018; 3D Systems Manufacturing, Rock Hill, SC, USA)	Deviations in RMS (A) 8 mm post space length: (1) PS: 0.33 ± 0.09 mm (2) Medit i500: 0.18 ± 0.03 mm (3) CS: 0.31 ± 0.07 mm (B) 10 mm post space length: (1) PS: 0.20 ± 0.07 mm (2) Medit i500: 0.18 ± 0.06 mm (3) CS: 0.26 ± 0.11 mm Post-scan length vs. post space depth (mean difference) (A) 8 mm post space length: (1) PS: −0.04 mm (2) Medit i500: −0.03 mm (3) CS: −1.42 mm (B) 10 mm post space length: (1) PS: −0.12 mm (2) Medit i500: −1.11 mm (3) CS: −3.53 mm	Trueness: 8 mm post length: Medit i500 > CS 3600 > Primescan 10 mm post length: Medit i500 > Primescan > CS 3600 Post space depth recording: 8 mm post length: Medit i500 > Primescan > CS 3600 10 mm post length: Primescan > Medit i500 > CS 3600
Almalki et al., 2023, USA [39]	In vitro	45	Typodont maxillary central incisors	Length: 6 mm 8 mm 10 mm Diameter: Size 4 Gates Glidden	CEREC Primescan IOS (Dentsply Sirona)	Conventional silicone impressions scanned using a lab scanner (inEos X5, Dentsply Sirona)	(A) Coronal third (B) Middle third (C) Apical third	Trueness	No	Superimposition	Geomagic Control X; 3D Systems	Deviations in RMS (i) 6 mm (A) Coronal third: 43 µm (B) Middle third: 64 µm (C) Apical third: 96 µm (ii) 8 mm (A) Coronal third:39 µm (B) Middle third:74 µm (C) Apical third: 131 µm (iii) 10 mm (A) Coronal third:55 µm (B) Middle third:80 µm (C) Apical third: 168 µm Overall RMS deviations: (i) 6 mm: 58 µm (ii) 8 mm:81 µm (iii) 10 mm:101 µm	Accuracy consistent in the coronal and middle third, irrespective of post length Accuracy decreases significantly in the apical third of longer post length (10 mm) Clinically acceptable impression with IOS for post spaces of 6 and 8 mm
Dupagne et al., 2023, France [40]	In vitro	1 (scanned 80 times)	3D printed model simulating mandibular premolars	Length: 10 mm Width at entrance: 3 mm	(1) Primescan, Dentsply Sirona, 5.2.2 (2) Omnicam, Dentsply Sirona, 5.2.2 (3) Medit i700, Medit Corp, 2.6.2 (4)TRIOS 4, 3Shape A/S, 21.4	Scan using E3 (Tabletop scanner) 3Shape A/S 2.1.6.1	Three depth ranges: (A) 0–3 mm (B) 3–6 mm (C) 6–9 mm (i) models with adjacent teeth (ii) Models without adjacent teeth	Mean measurement error (μm) at the three different depths	No	Superimposition	Metrological software (Gom inspect)	Mean measurement error (μm) (i) Models with adjacent teeth (1) Primescan: (A) 0–3 mm: 13.7 ± 0.4, (B) 3–6 mm: 12.7 ± 0.5, (C) 6–9 mm: 19.5 ± 2 (2) Omnicam: (A) 0–3 mm: 24.3 ± 0.5, (B) 3–6 mm: NE, (C) 6–9 mm: NE (3) TRIOS 4: (A) 0–3 mm: 26.4 ± 1.3, (B) 3–6 mm: 20.3 ± 2.1, (C) 6–9 mm: 25.7 ± 2.0 (4) Medit i700: (A) 0–3 mm: 24.0 ± 1.9, (B) 3–6 mm: 19.5 ± 0.7, (C) 6–9 mm: 22.3 ± 0.8 (5) E3 scanner: (A) 0–3 mm: 27.6 ± 0.6, (B) 3–6 mm: 28.2 ± 10, (C) 6–9 mm: NE (ii) Models without adjacent teeth (1) Primescan: (A) 0–3 mm: 13.7 ± 0.4, (B) 3–6 mm: 12.7 ± 0.5, (C) 6–9 mm: 19.5 ± 2 (2) Omnicam: (A) 0–3 mm: 14.6 ± 0.5, (B) 3–6 mm: 15.4 ± 0.8, (C) 6–9 mm: 19.2 ± 3.2 (3) TRIOS 4: (A) 0–3 mm: 18.4 ± 0.6, (B) 3–6 mm: 17.1 ± 0.8, (C) 6–9 mm: 15.9 ± 1.3 (4) Medit i700: (A) 0–3 mm: 12.4 ± 0.7, (B) 3–6 mm: 9.8 ± 0.5, (C) 6–9 mm: 16.4 ± 1.2 (5) E3 scanner: (A) 0–3 mm: 21.7 ± 3.2, (B) 3–6 mm: 24.0 ± 5.9, (C) 6–9 mm: NE	The presence of adjacent teeth can negatively affect the quality of root canal preparation digitized with IOSs Differences between different scanners. Overall, the lowest error was obtained using the Primescan IOS Tabletop scanner not an adapted tool for the digitizing of root canal preparation
Elter et al., 2022, Turkey [35]	In vitro	1 (scanned 8 times)	Natural extracted mandibular canines	Length: 20 mm 18 mm 16 mm 14 mm 12 mm 10 mm Diameter: 2.2 mm	CEREC Primescan 5.0.0 (Dentsply Sirona, Bensheim, Germany)	Conventional silicone impressions scanned using a lab scanner (CEREC inEos X5; Dentsply Sirona, Bensheim, Germany)	-	Trueness	No	Superimposition	Geomagic Control X 2020; 3D Systems; Rock Hill, SC, USA	Median RMS (i) 10 mm: 357.1 μm (ii) 12 mm: 419.6 μm (iii) 14 mm: 502.6 μm (iv) 16 mm: 528.3 μm (v) 18 mm: 856.2 μm (vi) 20 mm: 897.5 μm	Increase in post depth → increase in RMS Scanned space depth affected the trueness of IOS scans Post space depth > 14 mm → inadequate adaptation of the custom post and core → increased cement thickness IOS considered for impression-making for post space depth < 14 mm and diameter > 2.2 mm
Leven et al., 2022, Germany [36]	In vitro	5 (180 scans)	Natural extracted first mandibular premolars	Length: 12 mm Diameter: 0.9 mm	(1) Primescan (PS) (5.1, Dentsply Sirona, Bensheim, Germany) (2) Trios 4 (TRI) (19.2.4, 3Shape, Copenhagen, Denmark) (3) Trios 4 with Scan Post (TRI+SP)	Micro-CT (Skyscan 1173, Bruker Optics, Billerica, MA, USA)	(A) Apical, (B) First root canal third (C) Second root canal third (D) Occlusal internal (E) Occlusal external	Accuracy	Both with and without	Superimposition	3D analysis software GOM Inspect (version V8 SR1 2020, GOM Inspect, GOM GmbH, Braunschweig, Germany)	Linear discrepancy between intraoral scans and micro-CT (μm) # (A) Apical (1) PS: 55 (2) TRI: 55 (3) TRI+SP: 51.25 (B) First root canal third (1) PS:20.94 (2) TRI:25.94 (3) TRI+SP:23.75 (C) Second root canal third (1) PS:17.19 (2) TRI:14.06 (3) TRI+SP:14.38 (D) Occlusal internal: (1) PS:9.38 (2) TRI:15 (3) TRI+SP:12.81 (E) Occlusal external: (1) PS:25 (2) TRI:15 (3) TRI+SP:14.06	Deviations were within a clinically acceptable range The use of Scan Posts is not required for the current IOS and results in a lower accuracy
Kanduti et al., 2021, Slovenia [5]	In vitro	10	Natural extracted maxillary central incisors	Length: 6 mm Diameter: Size 2 Gates Glidden	TRIOS intraoral scanner (3Shape)	Conventional cast post (fabricated using pattern resin) and scanned using a laboratory scanner (Identica T300, Medit)	(A) Space volume (i) Cervical (ii) Middle (iii) Apical (B) Distance between the post and the prepared root canal wall among posts.	Accuracy	Yes Scan Post (1.7 APL, 3Shape)	Computed microtomography (μCT) Superimposition	Inspect, GOM	Mean and SD (A) Space volume: (a) Conventional cast post: (i) Coronal third: 0.57 ± 0.22 mm^3^ (ii) Middle third: 0.50 ± 0.27 mm^3^ (iii) Apical third: 0.41 ± 0.26 mm^3^ (b) Fully digital protocol: (i) Coronal third: 0.80 ± 0.19 mm^3^ (ii) Middle third: 1.07 ± 0.28 mm^3^ (iii) Apical third: 1.38 ± 0.41 mm^3^ (B) Distance between the post and the prepared root canal wall: (a) Conventional cast post: (i) Cervical section: 60.72 ± 32.51 μm and 34.33 ± 17.60 μm (ii) Apical section: 36.76 ± 34.05 μm and 85.73 ± 63.63 μm (b) Fully digital protocol: (i) Cervical section: 71.74 ± 25.51 μm and 41.99 ± 20.35 μm (ii) Apical section: 122.68 ± 27.40 μm and 185.60 ± 44.18 μm Overall (A) Space volume (a) Conventional cast post: 2.22 ± 1.35 mm^3^ (b) Fully digital protocol: 3.82 ± 0.45 mm^3^ (B) Distance between the post and the prepared root canal wall: (a) Conventional cast post: 53.66 ± 23.39 μm; (b) Fully digital protocol: 89.47 ± 19.30 μm. (B) Distance between the post and the prepared root canal wall: (a) Conventional cast post: 53.66 ± 23.39 μm (b) Fully digital protocol: 89.47 ± 19.30 μm	Space volume values in all measured sections: digital protocol > conventional approach The mean distance and the space area between the post and the prepared root canal wall in the apical sections: digital protocol > conventional approach No significant differences in cervical sections Overall accuracy: Conventional technique > digital protocol
Hendi et al., 2019, Iran [37]	In vitro	30	Natural extracted first and second mandibular premolars	Length: 10 mm Diameter: Size 2 Peeso Reamer	TRIOS 3D intraoral scanner (3Shape)	(A) Conventional resin pattern cast with cobalt-based alloy (B) Conventional silicone impressions scanned using a lab scanner (D700 Scanner, 3Shape) and Co–Cr alloy post using fabricated milling (half digital)	-	Apical gap	Yes	Parallel digital radiography	Ruler tool in image editing software (Adobe Photoshop CC; Adobe)	Apical gap: Conventional: 0.11 ± 0.06 mm Half digital: 0.66 ± 0.19 mm Fully digital: 0.29 ± 0.12 mm	Apical gap: Conventional < fully digital < half digital
Pinto et al., 2017, Italy [41]	In vitro	6	Natural extracted premolars	Length: 8.5–9.8 mm	TRIOS (3Shape)	Conventional silicone impressions; post length measured with millimeter gauge.	-	Reading depth	No	Manual	-	Mean depth discrepancy: Digital vs. conventional: 19.58% (5.92% to 37.94%)	Post space depth recording accuracy: Conventional technique > digital technique IOS has limitations in narrow root canals

RMS: Root Mean Square; IOS: Intraoral scanner; NE: Not exploitable; PS: Primescan; and #: Data retrieved using PlotDigitizer software, 3.1.6, 2024.

## Data Availability

The data that support the findings of this study are available from the corresponding authors upon reasonable request.

## Supplementary Materials

The following supporting information can be downloaded at: https://www.mdpi.com/article/10.3390/diagnostics14242890/s1, Supplementary File S1. PRISMA 2020 abstract checklist. Supplementary File S2. PRISMA 2020 checklist. Supplementary File S3. QUADAS-2 Table S1. Quality Assessment (QUADAS-2) summary of Risk Bias and Applicability concerns.
