# Supplementary material for: Dimensional Accuracy of Intraoral Scanners in Recording Digital Impressions of Post and Core Preparations: A Systematic Review
Source: Diagnostics (Basel). 2024 Dec 23;14(24):2890. doi: 10.3390/diagnostics14242890 (PMC11675224; doi:10.3390/diagnostics14242890)
Supplement: Supplementary file 1 [file diagnostics-14-02890-s001.zip › Supplementary file S3 QUADAS-2 Table S1 .pdf]

Table S1. Quality Assessment (QUADAS-2) summary of Risk Bias and Applicability concerns

Study	RISK OF BIAS				APPLICABILITY CONCERNS		
	PATIENT SELECTION	INDEX TEST	REFERENCE STANDARD	FLOW AND TIMING	PATIENT SELECTION	INDEX TEST	REFERENCE STANDARD
Taha et al, 2024 [12]	☺	☹	☺	☺	☺	☺	☺
Emam et al, 2023 [38]	☺	☺	☹	☺	☺	☺	☺
Almalki et al, 2023 [39]	☺	☹	☺	☺	☺	☺	☺
Dupagne et al., 2023 [40]	☺	☹	☺	☺	?	☺	☺
Elter et al, 2022 [35]	☺	☹	☺	☺	☺	☺	☺
Leven et al, 2022 [36]	☺	☹	☺	☺	☺	☺	☺
Kanduti et al, 2021 [5]	☺	?	?	☺	☺	?	?
Hendi et al, 2019 [37]	☺	☹	☺	☺	☺	☺	☺
Pinto et al., 2017 [41]	☺	☺	☹	☺	☺	?	?

☺ Low Risk ☹ High Risk ? Unclear Risk
